# Autism and Cortical Thickness Deviation From Neurotypical Controls: Evidence for a Spatial Association With Serotonin Receptors

**DOI:** 10.1002/aur.70243

**Published:** 2026-04-01

**Authors:** Livio Tarchi, Arne Doose, Julius Hennig, Fabio Bernardoni, Joseph A. King, Tiziana Pisano, Giovanni Castellini, Valdo Ricca, Inge Kamp‐Becker, Stefan Ehrlich

**Affiliations:** ^1^ Psychiatry Unit, Department of Health Sciences University of Florence Florence Italy; ^2^ Division of Psychological and Social Medicine and Developmental Neurosciences, Translational Developmental Neuroscience Section, Faculty of Medicine Technische Universität Dresden Dresden Germany; ^3^ German Center for Child and Adolescent Health (DZKJ), Partner Site Dresden/Leipzig Dresden Germany; ^4^ Neuroscience Department Meyer Children's Hospital IRCCS Florence Italy; ^5^ Department of Child and Adolescent Psychiatry Heidelberg University Hospital Heidelberg Germany

**Keywords:** autism, mental health, neurodevelopment, neurotransmitter receptors, structural neuroimaging

## Abstract

Cortical thickness (CT) differences between autistic individuals (AI) and neurotypical controls have been consistently reported, yet the neurochemical mechanisms underlying these differences remain insufficiently understood. Neurotransmitter receptor systems exhibit distinct spatial distributions across the cortex and influence synaptic maturation, plasticity, and cortical organization. Consequently, mapping CT deviations onto these receptor density gradients provides a biologically informed framework for investigating the potential underlying neurochemical architectures of observed structural patterns in autism. A total of 1035 structural MRIs (AI *n* = 505, neurotypical controls *n* = 530) were included from Autism Brain Imaging Data Exchange (ABIDE). Group contrasts and individual‐level deviations from age‐predicted CT patterns were computed. These spatial patterns were correlated with neurotransmitter receptors cortical density distributions (D1, D2, 5HT1a, 5HT2a, 5HT4, 5HT6, mGluR5) from previous PET studies. Widespread vertex‐wise deviations in CT were observed in AI in comparison to neurotypical controls. At the group level, CT differences were spatially aligned with the cortical density of serotonin receptors (5HT1a: *r* = 0.22, FDR‐*p* = 0.032; 5HT4: *r* = 0.21, FDR‐*p* = 0.032). At the individual level, greater deviations from predicted CT, if mapped onto specific neurotransmitter receptor density gradients, correlated with greater difficulties in the social and communication domains. The findings provide novel evidence that serotonin receptors may play a role in shaping cortical structural brain differences between AI and neurotypical controls. The link between CT differences and spatial distributions of the serotoninergic system offers a translational perspective for future targeted support strategies focused on serotonergic pathways.

## Lay Summary

Autistic individuals often have diverging brain structure compared to neurotypical controls, especially in regions linked to the neurotransmitter serotonin. In this study, these brain differences were associated with differences in social and communication skills. These findings show that serotonin may play a key role in autism and could help guide new support strategies.

## 1 Introduction

Autism is a neurodevelopmental condition defined by the DSM‐5‐TR as involving persistent differences in reciprocal social communication and interaction compared to neurotypical peers, alongside restricted, repetitive patterns of behavior or interests (American Psychiatric Association 2022). Globally, over 28 million individuals have been estimated to potentially meet diagnostic criteria for autism (Kang et al. 2023). The significant global burden associated with autism has been addressed by a World Health Assembly resolution, which called for strengthened research efforts to develop improved supportive strategies (World Health Organization, Executive Board 2013) in light of the relevant and often unmet needs of autistic individuals (AI), their families, and their peers.

Several studies have explored differences in brain morphology in AI, including: structural asymmetry (Kurth et al. 2024), surface area (Berg et al. 2023; Pretzsch et al. 2024; Yang et al. 2016), cortical volume (Ecker et al. 2013; Mei et al. 2024; Yang et al. 2016), subcortical volume (Zhang et al. 2018), gyrification (Yang et al. 2016), and cortical thickness (CT) (Doyle‐Thomas et al. 2013; James et al. 2022, 2025; Khundrakpam et al. 2017; Levman et al. 2019; Pretzsch et al. 2024; Richter et al. 2015; Shen et al. 2024; van Rooij et al. 2018; Yang et al. 2016; Zielinski et al. 2014). Of relevance for this study is CT, a morphological metric that serves as a macroscopic proxy for underlying microarchitectural features, such as synaptic density and dendritic arborization (Frangou et al. 2021). Given evidence suggests that AI exhibit widespread CT differences in comparison to neurotypical controls (both reductions and increases, depending on the specific hemisphere and brain region), and that the degree of CT difference may be correlated with reported difficulties within diagnostic domains for autism (Khundrakpam et al. 2017).

Other studies highlighted diverging patterns of development and cortical maturation in AI (Braden and Riecken 2019; Nunes et al. 2020; Pretzsch et al. 2024). Although recent studies described an association between atypical patterns of CT maturation and genes expressed during neurodevelopment (Ecker et al. 2022; Pretzsch et al. 2024; Romero‐Garcia et al. 2019), the mechanisms underlying CT structural differences in AI are not completely understood. However, a molecular‐informed approach may yield useful insight over potential influences of specific neurotransmitters underlying observed CT differences, considering how a preliminary report described a relationship between CT and neuroreceptor binding profiles across both AI and neurotypical controls (James et al. 2022).

In general, serotonin, dopamine, and glutamate have been among the most extensively studied neurotransmitters in relation to autism (Eissa et al. 2018; Marotta et al. 2020). In particular, serotonin appears to play a crucial role in several core characteristics associated with autism (Muller et al. 2016) and has been implicated in atypical neurodevelopmental trajectories in AI (Yang et al. 2014). In parallel, dopamine signaling has been suggested to play a role in autism and accompanying clinically relevant features (Eissa et al. 2018; Pavăl 2017), as well as being associated with autistic‐like traits in neurotypical controls (Pavăl 2017). Glutamate modulates synaptic plasticity at a cellular level and learning and memory at a behavioral one (Collingridge and Abraham 2022). Both synaptic plasticity and learning or memory have been described as different in AI (Boucher and Anns 2018; Kim and Kasari 2023; Zoghbi and Bear 2012).

A focus on the role of neurotransmitter receptors in autism may facilitate the development of translational approaches that link biological targets to—potential—clinically relevant distress experienced by some AI. The investigation of neurobiological mechanisms underlying clinical presentations and variability in autistic characteristics is an important goal of current research, as the absence of pharmacological options targeting core difficulties constitutes a present challenge in providing care for help‐seeking AI. In fact, pharmacological options currently only focus on the management of co‐occurring clinical features or co‐occurring conditions (Lord et al. 2018), a limitation explicitly mentioned by current clinical guidelines (Crowe and Salt 2015).

In particular, antipsychotic medications (i.e., Risperidone, Aripiprazole) are usually employed to target agitation or irritability, while stimulants (i.e., Methylphenidate) may be adopted in case of co‐occuring diagnoses of ADHD (Lord et al. 2018). Selective serotonin‐reuptake inhibitors (SSRI) are also commonly prescribed to AI, usually to target depression or anxiety (Rai et al. 2024). While the neurobiological mechanism of action of most psychiatric medications is complex (Johansen et al. 2023), SSRIs, antipsychotic medications, and stimulant medications exert at least part of their effect through the modulation of the serotonin, dopamine and glutamate neurotransmitter systems at a central level (Lerond et al. 2013; Liu et al. 2006; MacQueen et al. 2001; S. Wang et al. 2018). For these reasons, the current work attempts to explore whether CT differences in AI may be spatially aligned with the underlying cortical distribution and density of these neurotransmitter receptors.

Indeed, while multiple biological systems contribute to the molecular underpinnings of brain organization, the present study focuses on neurotransmitter systems for both conceptual and methodological reasons. Previous investigations of biological correlates of cortical organization have largely relied on region‐of‐interest‐based approaches, limiting spatial specificity and constraining inferences about continuous cortical variation (Ecker et al. 2022; James et al. 2022; Pretzsch et al. 2024; Romero‐Garcia et al. 2019). In contrast, neurotransmitter system characteristics as measured by PET currently represent one of the few classes of relevant molecular features for which reference maps are available at a vertex‐wise resolution (Beliveau et al. 2017; Markello et al. 2022; from here on referred to as “chemoarchitecture” i.e., the spatial distribution of these signatures). In parallel, recent methodological advances have enabled reliable transformations between PET‐ and MRI‐derived data across stereotactic spaces, allowing direct spatial correspondence between in vivo neuroimaging measures and molecular reference maps (Markello et al. 2022).

As such, neurotransmitter system characteristics offer a biologically grounded and technically feasible entry point for vertex‐wise investigations of molecular–macroscale brain relationships. Importantly, this focus does not imply that neurotransmitters are the sole or primary biological mechanisms of interest. Rather, it reflects current constraints in the availability of vertex‐resolved reference data for other biological correlates, such as gene expression, cellular composition, or metabolic markers, many of which would require distinct acquisition modalities and pose substantial technical challenges for integration.

### 1.1 Aims

The primary aim of the study was to investigate how the spatial distribution of CT differences in AI in comparison to neurotypical controls may be shaped by the underlying chemoarchitecture of the brain (i.e., the cortical density of specific neurotransmitter receptors). For this reason, vertex‐wise group differences of CT were first estimated. The resulting group contrast was compared with the spatial density maps of serotonin, dopamine and glutamate receptors, based on a number of published PET studies retrieved by *neuromaps* (Markello et al. 2022).

The secondary objective of the study was to investigate whether the level of spatial overlap between individual deviations from predicted CT (at the vertex‐level) and chemoarchitecture features would be associated with reported difficulties in the domains of communication, social interaction, or the presence of repetitive, stereotyped behaviors in AI.

## 2 Materials and Methods

### 2.1 Sample and Participants

A total of 1035 participants (autistic participants *n* = 505, neurotypical controls *n* = 530) were included in the present study after quality control (excluded individuals; *n* = 77, of which 34 AI and 43 neurotypical controls). The data were retrieved from the public dataset published by Autism Brain Imaging Data Exchange (ABIDE) consortium, which collected psychometrics, anthropometrics, structural MRI, and functional neuroimaging measures across 20 different sites (Cameron et al. 2013; Di Martino et al. 2014). The study protocols were ethically approved by all local Institutional Review Boards, and all participants (and, if minors, their legal guardians) gave written informed consent. Further information about the study can be found on the ABIDE website (http://fcon_1000.projects.nitrc.org/indi/abide/, including phenotypic data and quality assessment information http://s3.amazonaws.com/fcp‐indi/data/Projects/ABIDE_Initiative/Phenotypic_V1_0b_preprocessed1.csv). See the original documentation for further details on neuroimaging data acquisition.

### 2.2 Clinical and Psychometric Evaluation

Throughout ABIDE centers, clinical DSM‐IV diagnoses of autism were made by combining clinical judgment and Autism Diagnostic Observation Schedule (ADOS) and the Autism Diagnostic Interview‐Revised (ADI) (for further information see Zhang et al. 2018; Di Martino et al. 2014). Both assessments consist of three subscales, namely: language and communication (C); reciprocal social interaction (RSI); stereotyped behaviors/restricted interests (RRB). Higher scores represent higher levels of difficulties. A total of 357 ADI scores and 351 ADOS scores were retrieved for included individuals with autism. ADI scores were not available for neurotypical controls, and thus no group comparison was estimated. Medication status was known for 379 included AI out of 505 (75.05%; of which 254 were not medicated and 125 medicated).

### 2.3 CT Estimation

#### 2.3.1 Structural MRI

The ABIDE consortium offers preprocessed individual structural MRI images, which were analyzed by Freesurfer 5.1 (Cameron et al. 2013). Thus, the estimated total intracranial volume (ICV) and vertex‐wise CT were retrieved. ABIDE preprocessed individual structural MRI data have been regularly studied due to the large sample size and easy availability (Díaz‐Caneja et al. 2019; Hong et al. 2019; King et al. 2020). Some studies described harmonization efforts in order to enhance the accuracy of diagnostic algorithms based on CT (Ingalhalikar et al. 2021; Saponaro et al. 2022). However, these methods leveraged ROI‐based results (Ingalhalikar et al. 2021; Saponaro et al. 2022), thus employing standard harmonizing techniques such as neuroCombat (Fortin et al. 2018). However, its application for vertex‐wise maps is less standardized, and test–retest studies showed that CT may show negligible variability within scanners, between scanners, and between field strengths at the sample size reached by the ABIDE consortium (Han et al. 2006). For the aforementioned reasons, non‐harmonized vertex‐wise data were used in the primary analysis. Nevertheless, to rigorously control for potential site‐related confounds, site effects were explicitly modeled in a robustness check by including site indicator variables as covariates in the general linear model.

#### 2.3.2 Group Contrast

FreeSurfer's general linear model was employed to calculate the statistical difference between AI and neurotypical controls for vertex‐wise CT, with age and sex as a covariate (see Equation 1).

(1) $$\hat{\mathrm{CT}}=\beta_{0}+\beta_{1}*\mathrm{age}+\beta_{2}*\mathrm{sex}+\beta_{3}*\text{group}+e$$

where the third coefficient represented the group contrast.

Moreover, to account for nonlinear developmental trajectories that a linear model might miss, we repeated the analysis while also including a quadratic age term (age^2^) in the model (Equation 2).

(2) $$\hat{\mathrm{CT}}=\beta_{0}+\beta_{1}*\mathrm{age}+\beta_{2}*{\mathrm{age}}^{2}+\beta_{3}*\mathrm{sex}+\beta_{4}*\text{group}+e$$

#### 2.3.3 Individual‐Level Spatial Distribution of CT Deviation

Individual‐level residual errors were defined as the difference to the vertex‐wise predicted CT value, as a function of age and sex. The term *e* in Equation (1) represents this measure. This individual residual error was interpreted as estimate of an individual's deviance from sex‐ and age‐predicted CT. In order to assess the individual‐level spatial distribution of CT deviations, individual‐level residual errors were thus first extracted and then correlated with the spatial distribution of neurotransmitter receptors, see Statistical analysis section for further details. The choice to rely on FreeSurfer's general linear models was based on the need to retain vertex‐wise information, without imposing additional dimensionality reduction or prior recalibration steps (e.g., as in pretrained normative Bayesian models), thereby minimizing potential sources of bias.

#### 2.3.4 Cortical Density of Neurotransmitters—Reference Feature Maps

The Python package *neuromaps* is a collection of methods and maps which allows for comparison of empirically measured maps (e.g., CT) with a selection of reference feature maps (i.e., spatial density of neurotransmitter receptors or transporters, based on radioactive ligand binding studies). In light of genetic, pharmacological and molecular evidence of neurotransmitter system differences in AI in comparison to neurotypical controls (Hervás et al. 2014; Nisar et al. 2022; Pavăl 2017; Rodnyy et al. 2024), the following reference chemoarchitecture features were retrieved (*neuromaps* atlases from PET binding studies): dopamine receptors (D1, D2), serotonin receptors (5HT1a, 5HT1b, 5HT2a, 5HT4, 5HT6), glutamate receptors (metabotropic glutamate receptor 5‐mGluR5) (Hansen et al. 2022; Markello et al. 2022). For more details on reference feature maps, see eMethods S1.

### 2.4 Statistical Analysis

#### 2.4.1 Spatial Association of Group‐Level CT Differences With Chemoarchitecture Features

To estimate and test the spatial alignment between group‐level results and chemoarchitecture features (i.e., neurotransmitter receptor densities), *neuromaps* was first employed to transform data to the native space of choice (fsaverage). In brief, as previously mentioned, *neuromaps* uses standard tools (Buckner et al. 2011; Robinson et al. 2014, 2018; Wu et al. 2018) in order to perform the transformation between surfaces, volumes or from volumes to surfaces. Surface‐to‐surface transformations were used to convert the reference feature maps to fsaverage. Once transformed to fsaverage, *neuromaps* was used to estimate the vertex‐wise spatial correlation (Pearson's *r*) between the two images across the whole surface (Markello et al. 2022). Null models were then computed according to BrainSMASH method (Burt et al. 2020), which generates surrogate maps by permitting spatial autocorrelation to be preserved through variogram matching based on a distance matrix, rather than simple rotation, to derive the null distribution of correlation coefficients (spatial nulls, *n* = 1000). See eMethods S2 for a graphical representation of study procedures and analysis workflow.

#### 2.4.2 Spatial Association of Individual‐Level CT Deviations With Chemoarchitecture Features

Similarly to group‐level results, to estimate and test the spatial alignment between individual‐level results (i.e., individual level residuals—term *e* in Equation 1) and neurotransmitter densities, *neuromaps* was employed to transform data to the space of choice (fsaverage) and compute Pearson's *r* coefficients between the two images. Null models were then computed according to BrainSMASH method (Burt et al. 2020; spatial nulls, *n* = 1000).

#### 2.4.3 Clinical Correlates of Spatial Association Between Individual‐Level CT Deviations and Chemoarchitecture Features

Spatial association between observed CT differences and chemoarchitecture features were first computed for each AI (see “Spatial associations of individual‐level CT deviations with chemoarchitecture features”), then, Fisher‐Z transformed. This individual‐level fisher‐Z transformed score was finally correlated with psychometrics scores. To control for possible site effects such as rater‐dependent effects on psychometric results (Zander et al. 2016) and effects due to scanner characteristics, these correlation analyses were limited to a single center (New York University—NYU, the site with the largest sample size, including 71 AI after quality control). Correlations with psychometric (i.e., clinical) scores were computed with ADOS and ADI scores in the group of AI (C, RSI, RRB, ADOS total score). Correlation coefficients were estimated within AI only, after age and sex adjustment (partial r). Confidence intervals were bootstrapped (*n* = 5000) in order to estimate the reliability of reported confidence intervals.

#### 2.4.4 Robustness Checks

As a robustness check, we conducted two additional analyses. First, we repeated the primary analysis using a more rigorous image quality assessment, excluding any participant rated as “poor” by at least one rater (resulting in the further exclusion of 115 individuals, of whom 71 were AI and 44 were neurotypical controls). Second, to control for site effects, we additionally included site as a covariate. Due to the high number of scanning sites relative to the sample size (and the limited number of female participants in certain centers), site effects were modeled using dummy coding rather than complex hierarchical interactions (see Equation 3).

(3) $$\hat{\mathrm{CT}}=\beta_{0}+\beta_{1}*\mathrm{age}+\beta_{2}*{\mathrm{age}}^{2}+\beta_{3}*\text{group}+\beta_{\text{site}}+e$$

Clinical correlates were also re‐computed after controlling for the role of IQ. This was done to ascertain whether the correlation between ADOS and ADI scores (C, RSI, RRB, ADOS total score) with these features could be biased by IQ.

## 3 Results

The group of neurotypical controls had a larger proportion of females than the group of AI (Chi‐Squared = 6.409; *p* = 0.011), but did not differ in terms of age. Autistic participants exhibited significantly higher symptom scores in all subscales as assessed by ADOS in comparison to neurotypical controls (Table 1).

**TABLE 1:** Sample descriptives of included participants (*n* = 1035).

	AI (*n* = 505)	TYP (*n* = 530)	U‐score/Chi‐squared	*p*
Demographics				
Age (in years)	17.10 ± 8.55	16.81 ± 7.45	132,751	0.823
Sex	Males: 443, females: 62	Males: 435, females: 95	6.409	0.011
BMI	21.50 ± 5.84	19.50 ± 4.49	1180	0.243
Intracranial volume (mm^3^)	1,328,967 ± 274,413	1,301,948 ± 253,910	142,096	0.075
Psychometric scores				
ADI C	15.70 ± 4.65	/	/	/
ADI RSI	19.71 ± 5.55	/	/	/
ADI RRB	6.04 ± 2.56	/	/	/
ADOS C	3.79 ± 1.56	0.50 ± 0.61	6406	< 0.001
ADOS RSI	8.06 ± 2.71	0.65 ± 0.81	6567	< 0.001
ADOS RRB	2.10 ± 1.54	0.20 ± 0.70	4751	< 0.001
ADOS total score	11.80 ± 3.77	1.15 ± 1.14	7013	< 0.001

*Note*: Mean value±standard deviation for each variable and study group are shown. As data were not normally distributed, group differences were tested using Mann–Whitney two sample *t*‐tests. As test statistics, *U*‐score and *p*‐values are stated. Chi‐squared was calculated for sex. Autism Diagnostic Interview‐Revised (ADI) scores were not available for neurotypical controls. Further details on subsamples by site of acquisition can be found at http://preprocessed‐connectomes‐project.org/abide/.

Abbreviations: ADI C = ADI communication; ADI RRB = ADI restricted, repetitive and stereotyped patterns of behavior; ADI RSI = ADI reciprocal social interaction; ADOS C = ADOS communication; ADOS RRB = ADOS repetitive and stereotyped patterns of behavior; ADOS RSI = ADOS reciprocal social interaction; ADOS = Autism Diagnostic Observation Schedule; AI = autistic individuals; TYP = neurotypical controls.

### 3.1 Spatial Association of Group‐Level CT Differences With Chemoarchitecture Features

In accordance with previous evidence collected in the ABIDE dataset (Khundrakpam et al. 2017), widespread greater vertex‐wise CT was observed in AI in comparison to neurotypical controls. See Supporting Information Figure S1 for a graphical representation of the present group contrast.

The distribution of CT differences in AI showed significant correlations with reference features of chemoarchitecture maps. In particular, the CT group contrast map was significantly correlated with the vertex‐wise spatial density of 5HT1a and 5HT4 receptors (5HT1a: Pearson's *r* = 0.22, FDR‐*p* = 0.032; 5HT4: *r* = 0.21, FDR‐*p* = 0.032, Benjamini‐Hochberg corrected). Regions with greater CT in the group of AI compared to neurotypical controls were specifically associated with brain areas expressing higher densities of 5HT1a and 5HT4 receptors, while regions with lower CT were associated with areas expressing lower densities of these receptors.

**FIGURE 1:**
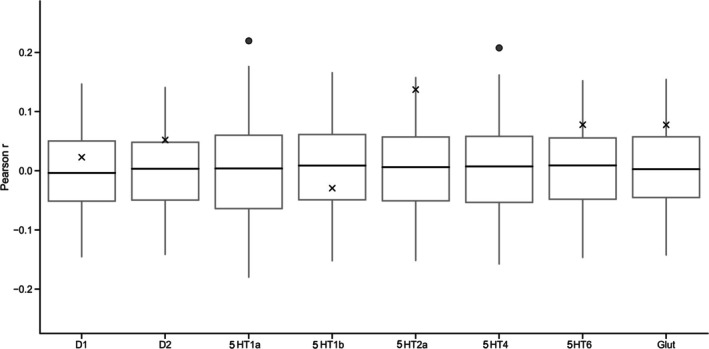
Spatial associations of group‐level cortical thickness differences with chemoarchitecture features. Boxplots representing correlation coefficients for rotated images (1.000 permutations), in order to represent 95% confidence intervals of null distributions (BrainSMASH; Burt et al. 2020). The analysis was controlled for the effect of age and sex. Empirical results are represented by an “*x*” if they are not statistically significant, and by a red point if statistically significant (FDR‐*p* < 0.05). Dopamine receptors (D1, D2), serotonin receptors (HT1a, HT1b, HT2a, HT4, 5HT6), glutamate receptors (Glut).

See Figure 1 for a graphical representation of this analysis, and Supporting Informations Figure S2a and Figure S2b for a visual representation of cortical regions involved both in CT differences and in the expression of specific neurotransmitter receptors. Further analyses in the same sample, controlling for quadratic effects of age, confirmed these results (5HT1a: Pearson's *r* = 0.36, FDR‐*p* = 0.040; 5HT4: *r* = 0.32, FDR‐p = 0.040; see Supporting Informations Figure S3 for a graphical representation of results).

### 3.2 Clinical Correlates of Spatial Association Between Individual‐Level CT Deviations and Chemoarchitecture Features

Next, we examined the association between clinical presentations (C, RSI, RRB) and the spatial alignment of individual neuroanatomy with underlying chemoarchitecture features, quantified as the correlation coefficients between individual‐level CT deviations and serotonin receptor density maps (see Supporting Informations Figure S4 for a graphical representation of selected representative individual maps). Only chemoarchitecture feature maps exhibiting significant correlations with CT differences at the group level were included (5HT1a, 5HT4). This correlation analysis was limited to the single research center with the greatest number of participants (NYU; *n* = 71 AI after quality control).

Greater deviance from age‐predicted CT in regions with a high density of 5HT1a and 5HT4 receptors positively correlated with ADI RSI and ADI C scores. Moreover, greater deviance in 5HT4‐related brain regions positively correlated with ADOS RSI and ADOS C, as well as ADOS total score, i.e., higher correlation coefficients of individual CT deviations and serotonin receptor density was linked to higher ADI and ADOS scores. See Table 2 for further details, Supporting Informations Figure S5a and Supporting Informations Figure S5b illustrate the results as scatter plots.

**TABLE 2:** Clinical correlates, Pearson's partial correlations (corrected for age and sex).

	5HT1a [95% CI]	5HT4 [95% CI]
ADI C	0.260 [−0.028, 0.466]	0.277* [0.028, 0.479]
ADI RSI	0.269 [−0.008, 0.474]	0.281 * [0.009, 0.507]
ADI RRB	0.086 [−0.192, 0.370]	0.069 [−0.196, 0.324]
ADOS C	0.143 [−0.088, 0.338]	0.302* [0.089, 0.497]
ADOS RSI	0.180 [−0.083, 0.438]	0.217 [−0.041, 0.468]
ADOS RRB	−0.150 [−0.344, 0.073]	−0.079 [−0.271, 0.145]
ADOS Total Score	0.186 [−0.061, 0.412]	0.274* [0.039, 0.493]

*Note*: 95% confidence intervals [CI] estimated by bootstrapping, 5000 resamplings.

Abbreviations: ADI C = ADI communication; ADI RRB = ADI restricted, repetitive and stereotyped patterns of behavior; ADI RSI = ADI reciprocal social interaction; ADI = Autism Diagnostic Interview‐Revised; ADOS C = ADOS communication; ADOS RRB = ADOS repetitive and stereotyped patterns of behavior; ADOS RSI = ADOS reciprocal social interaction; ADOS = Autism Diagnostic Observation Schedule.

* Bootstrapped *p* < 0.05.

### 3.3 Robustness Checks

First, the primary analysis was repeated using stricter quality control criteria while accounting for nonlinear age effects (Equation 2). A sample description after stricter quality control and the exclusion of an additional 115 participants can be found in Supporting Informations Table S1. See Supporting Informations Figure S6 for the simple group contrast for this robustness check. This control analysis confirmed the robustness of our results, specifically that both 5HT1a and 5HT4 distributions were spatially correlated with group‐level CT differences (5HT1a: Pearson *r* = 0.29, FDR‐*p* = 0.040; 5HT4: Pearson *r* = 0.27, FDR‐*p* = 0.040; Supporting Informations Figure S7).

Second, further analyses were conducted controlling for site effects (see Section 2, Equation 3). They were fully concordant with main results (5HT1a: Pearson *r* = 0.29, FDR‐*p* = 0.021; 5HT4: Pearson *r* = 0.29, FDR‐*p* = 0.021; Supporting Information Figure S8). Finally, correlations between individual‐level CT deviations within 5HT1a/5HT4 regions and ADI/ADOS scores were repeated in the NYU sample, adjusting for IQ in addition to sex and age. Significant correlations were also observed when applying this further correction (see Supporting Informations Table S2).

## 4 Discussion

The current study examined whether CT differences between AI and neurotypical controls align with the underlying spatial distribution of serotonin, dopamine, and glutamate neurotransmitter receptors throughout the cerebral cortex. The findings revealed that the pattern of CT differences in AI was spatially aligned with the distribution of specific serotonin receptors, in particular with the cortical distribution of 5HT1a and 5HT4 serotonin receptors. This suggests a potential underlying molecular component for structural brain differences, which have been widely reported in AI (Khundrakpam et al. 2017; Levman et al. 2019; Pretzsch et al. 2024; Shen et al. 2024; van Rooij et al. 2018; Yang et al. 2016).

Notably, this spatial association aligns with the hypothesis that the serotoninergic system may act as a key regulator of cortical development in brain differences observed between AI and neurotypical controls (Wegiel et al. 2024). Furthermore, at the individual level, autistic participants with greater CT in regions with a higher 5HT1 or 5HT4 receptor expression also exhibited more difficulties in the domains of communication and reciprocal social interaction (with medium effect sizes *r*~0.3). Taken together, these results suggest a potential association between serotonergic signaling pathways and characteristic behavioral patterns associated with autism and indicate that individual‐level profiles may help to account for phenotypic heterogeneity. This insight may help guide the identification of future novel pharmacological support strategies for AI. It should be noted, however, that in contrast with previous literature emphasizing the role of dopamine and glutamate in autism (Nisar et al. 2022; Pavăl 2017), the present study did not find any significant spatial correlation between CT differences and the receptor distribution of these neurotransmitters.

As previously mentioned, the present study builds on previously reported findings of widespread differences in vertex‐wise CT in AI compared to neurotypical controls (Khundrakpam et al. 2017; Shen et al. 2024; Zielinski et al. 2014). Earlier research in the ABIDE cohort highlighted primarily left lateralized CT differences (higher CT in the group of AI compared to neurotypical controls) (Khundrakpam et al. 2017). These previous results are partly aligned with those reported in a large‐scale mega‐analysis of CT differences as reported by the ENIGMA‐consortium, which found greater CT in lateral frontal regions, but also lower CT in temporal regions in AI (van Rooij et al. 2018). Discrepancies between these studies may be attributable to methodological differences: ABIDE studies employed vertex‐wise results, while ENIGMA studies adopted a region‐based approach—averaging CT values across left and right hemispheres (Khundrakpam et al. 2017; van Rooij et al. 2018). Averaging CT across left and right regions may mask group differences, as a significant asymmetry between divergences with neurotypical controls across the two hemispheres has been described in AI, especially for frontal and temporal regions (Postema et al. 2019).

As noted above, our main finding was that CT differences between AI and neurotypical controls were spatially aligned with selected serotonin receptors. 5HT1a receptors have been extensively investigated in animal models of autism (Rodnyy et al. 2024). These studies showed how 5HT1a receptors are under‐expressed in the hippocampus of mice models of autism, with evidence of phenotype reversal after the restoration of 5HT1a expression (Kondaurova et al. 2023). It has been suggested that pharmacological interventions based on 5HT1a agonist action may increase social interaction in animal models of autism (Wang et al. 2013)—although a full translation of these results to human individuals has yet to be undertaken. In brief, the present findings are in line with previous evidence from animal models indicating the role of the serotonergic system in the promotion of prosocial behavior, further supporting an association between the serotonin system and communication differences accompanying autism (Muller et al. 2016).

Similarly to 5HT1a, 5HT4 receptors have also been previously associated with core characteristics of autism, based on preliminary evidence suggesting that 5HT4 hypermethylation may be associated with a higher probability to be diagnosed with autism itself (Hu et al. 2020). Although downstream and regulatory mechanisms underlying the link between 5HT1a/5HT4 and clinical characteristics potentially accompanying autism remain elusive, some evidence suggests that the interactions between 5HT1a, other serotonin receptors, the TrkB pathway and BDNF may play a role (Kondaurova et al. 2023). Specifically, BDNF promotes synaptic stability, synaptogenesis and dendritogenesis, while also upregulating protective molecular pathways and enhancing overall neuronal survival (Bartkowska et al. 2007; Yoshii and Constantine‐Paton 2007).

In general, previous studies showed underlying differences in serotonin expression between AI and neurotypical controls (Cook et al. 1994; Muller et al. 2016). In fact, hyperserotonemia has been frequently proposed as a potential biomarker for autism, based on consistent findings of elevated peripheral serotonin levels in both AI and first‐degree relatives (Gabriele et al. 2014; Montgomery et al. 2018). This elevation in peripheral serotonin levels (i.e., hyperserotonemia) may reflect diverging serotonin expression in the central nervous system (Muller et al. 2016), and thus be linked to CT differences within specific brain regions in AI (Wegiel et al. 2024; Whitaker‐Azmitia 2005)—particularly those with high densities of specific serotonin receptors. Furthermore, it has been proposed that stronger downstream signals of serotonin via 5HT4 may be associated with CT differences—given the role of 5HT4 in regulating the functional maturation of dendritic spines and long‐term potentiation of excitatory transmissions (Ogelman et al. 2024; Schill et al. 2020). This described molecular cascade would link neurotransmitter synthesis (possibly related to hyperserotonemia) to neuroanatomical deviances, in light of the action of specific serotonergic receptors (Daly et al. 2014; Khundrakpam et al. 2017; Langen et al. 2012; Postema et al. 2019; Wegiel et al. 2024).

The aforementioned studies regarding peripheral serotonin levels (Cook et al. 1994; Muller et al. 2016) are also of interest in light of evidence indicating that reducing serotonin availability at the individual level can differently regulate core autistic characteristics (Daly et al. 2014). In line with these findings, our individual‐level analyses revealed that greater CT within cortical areas known to be characterized by higher density of 5HT1a or 5HT4 were associated with greater difficulties in the domains of reciprocal social interaction and communication. When controlling for IQ, CT differences in areas known to be characterized by a higher density of 5HT1a only correlated with scales reflecting clinical presentations during childhood (as measured by ADI). By contrast, greater CT in areas known to be characterized by a higher density of 5HT4 also correlated with scales reflecting clinical presentations at the time of evaluation with the study (as measured by ADOS). These preliminary results suggest that clinical correlates for 5HT1a receptor and CT distribution patterns may attenuate during development, while behavioral and clinical correlates for 5HT4 receptors may persist during adolescence and, possibly, adulthood. Future studies might explore whether individual‐level deviation from age and sex predicted CT, alongside related chemoarchitecture features, may help in delineating diverging biotypes in autism (Litman et al. 2025). Indeed, integrating molecular and/or neurochemical signatures to explain CT differences may provide critical insight into the substantial heterogeneity among AI (Mei et al. 2024; Xu et al. 2024). Future studies might extend our approach to transcriptomic features, as recently demonstrated in a methods report by Ecker et al. (2025).

Future studies could also explore personalized support strategies in light of specific biotypes and specific patterns of distribution of CT differences at the individual‐level. Refining these biotypes necessitates a nuanced understanding of biological sex, particularly in light of recent findings that the neuroanatomical correlates of specific clinical features, such as sensory processing, exhibit sex‐divergent pathways (James et al. 2025). Indeed, if corroborated by future longitudinal studies, current results suggest that restoring serotoninergic imbalances may be particularly beneficial for those individuals for whom differences in comparison to age‐ and sex‐predicted CT patterns spatially align to 5HT1a/5HT4 receptors—as the same individuals may potentially also report greater difficulties or distress in specific clinical domains. Empirical evidence lends support to this assumption, as 5HT1a/5HT4 agonists have been shown to ameliorate co‐occurring conditions and potentially target specific domains of distress in AI (Chugani et al. 2016; Ghanizadeh and Ayoobzadehshirazi 2015; Hollander et al. 2012). Moreover, in animal models, the degree of response to 5HT1a agonists appears to be influenced by individual factors, including 5HT1a gene upregulation (Gould et al. 2011), thereby providing further evidence that it may be possible to develop personalized support strategies in light of observed patterns of divergence for 5HT1a/5HT4 receptors.

### 4.1 Limitations

While this study provides new insight into promising neurobiological correlates of autism, several limitations should be considered. First, the cross‐sectional design limits the ability to establish causality or determine temporal relationships between CT differences, chemoarchitectonic features, and clinical presentations. Future longitudinal studies would enable a more comprehensive investigation of how individual‐level CT deviations associated with chemoarchitecture features may shape neurodevelopmental and potential clinical trajectories in AI. It is acknowledged that the utilization of non‐harmonized multi‐site data carries inherent limitations (Mei et al. 2024), where images were collected on different scanners with different parameters (Table 1). However, additional analyses suggest that the results remained robust when controlling for site effects. Both autistic participants and neurotypical controls were assessed across different stages of development. For this reason, additional analyses were conducted to control for the role of age (controlling for linear and quadratic effects). Further, atypical sensory responsiveness is a prominent feature of autism and has been previously linked to differences in CT in the visual, auditory, and somatosensory cortices (Habata et al. 2021). However, the present study could not examine associations with sensory responsiveness, as the ABIDE dataset does not include assessments of this domain. Future studies incorporating detailed and dimensional measures of sensory processing should explicitly investigate how sensory responsiveness relates to underlying chemoarchitectural features in autism. Individual‐level analyses using clinical correlates were not controlled for multiple comparisons. Therefore, although a consistent pattern of association with HT4 receptors emerged for communication difficulties across ADI and ADOS scores, these results should be considered exploratory in nature pending replication in independent samples. Last, but not least, the cortical densities of neurotransmitter receptors were taken from several previous studies (with limited sample sizes and adult participants) based on samples composed of neurotypical controls, potentially introducing bias in current results. This key limitation is shared with previously mentioned approaches—for instance, previous studies based on transcriptome reference data (Berg et al. 2023; Ecker et al. 2022, 2025; Pretzsch et al. 2024; Romero‐Garcia et al. 2019), which were also based on relatively small samples of neurotypical individuals (mainly adult males).

## 5 Conclusions

The present study proposes a link between CT deviances observed in AI compared to neurotypical controls and the spatial distribution of serotonin receptors within the brain (5HT1a, 5HT4). A similar link was not supported for receptors in the dopaminergic or glutamatergic systems. The degree of overlap between individual deviance from age‐predicted CT and serotonin receptors (5HT1a, 5HT4) was positively correlated with core difficulties reported by AI, particularly in domains related to reciprocal social interaction and communication. Together, these findings shed new light on our understanding of the complex neurobiology linking autism‐related CT differences, clinical presentations, and underlying chemoarchitectonic features. Current results may inform future development of personalized support strategies and new pharmacological interventions.

## Funding

Work supported by #NEXTGENERATIONEU (NGEU) and funded by the Ministry of University and Research (MUR), National Recovery and Resilience Plan (NRRP), project MNESYS (PE0000006)—a multiscale integrated approach to the study of the nervous system in health and disease (DN. 1553—DN. 11.10.2022). Funding included expenses related to personnel. No influence on research results was exerted by the funding agency. The project was funded by the Federal Ministry of Education and Research (Bundesministerium für Bildung und Forschung, BMBF) as part of the German Center for Child and Adolescent Health (DZKJ) under the funding code 01GL2405B and by the research consortium on Autism spectrum disorders, ASD‐Net (FKZ 01EE1409A).

## Ethics Statement

After receiving a verbal explanation of the study, all participants gave written informed consent following procedures approved by each local Institutional Review Board. Please refer to the documentation provided by the ABIDE consortium for further information http://preprocessed‐connectomes‐project.org/abide/.

## Consent

All participants gave written informed consent following procedures approved by each local Institutional Review Board. Please refer to the documentation provided by the ABIDE consortium for further information http://preprocessed‐connectomes‐project.org/abide/.

## Conflicts of Interest

The authors declare no conflicts of interest.

## Supporting information


**eMethods S1.** Provides further information on selected feature maps.
**eMethods S2**. Graphically illustrates study procedures and analysis workflow.
**Table S1:** Provides sample descriptives, after stricter MRI quality control.
**Figure S1:** Illustrates the group contrast for cortical thickness between autistic individuals and neurotypical controls, controlling for the effect of age and sex.
**Figure S2a:** Illustrates the overlap between the group contrast for cortical thickness between autistic individuals and neurotypical controls and the selected 5HT1a receptor density feature map.
**Figure S2b:** Illustrates the overlap between the group contrast for cortical thickness between autistic individuals and neurotypical controls and the selected 5HT4 receptor density feature map.
**Figure S3:** Illustrates the spatial association between the group contrast for cortical thickness between autistic individuals and neurotypical controls and selected feature maps, after controlling for age, sex and age^2^.
**Figure S4:** Illustrates representative individual maps of deviance from age and sex predicted cortical thickness.
**Figure S5a:** Illustrates the correlation between clinical values (ADI scores) and the spatial association—at the individual level—of cortical thickness deviations with serotonin receptor densities.
**Figure S5b:** Illustrates the correlation between clinical values (ADOS scores) and the spatial association—at the individual level—of cortical thickness deviations with serotonin receptor density.
**Figure S6:** Illustrates the group contrast for cortical thickness between autistic individuals and neurotypical controls, controlling for the effect of age, sex, age^2^, and after stricter quality control.
**Figure S7:** Illustrates the spatial association between the group contrast for cortical thickness between autistic individuals and neurotypical controls and selected feature maps, after controlling for age, sex, age^2^, and after stricter quality control.
**Figure S8:** Illustrates the spatial association between the group contrast for cortical thickness between autistic individuals and neurotypical controls and selected feature maps, after controlling for age, sex, age^2^, acquisition site, and after stricter quality control.

## Data Availability

The datasets generated during the current study, as well as the code supporting the analyses, are available from the corresponding author upon reasonable request. The original data can be retrieved from the ABIDE consortium http://preprocessed‐connectomes‐project.org/abide/.
