# Supplementary material for: Autism and Cortical Thickness Deviation From Neurotypical Controls: Evidence for a Spatial Association With Serotonin Receptors
Source: Autism Res. 2026 Apr 1;19(6):e70243. doi: 10.1002/aur.70243 (PMC13276705; doi:10.1002/aur.70243)
Supplement: Supplementary file 1 — eMethods S1. Provides further information on selected feature maps. eMethods S2. Graphically illustrates study procedures and analysis workflow. Table S1: Provides sample descriptives, after stricter MRI quality control. Figure S1: Illustrates the group contrast for cortical thickness between autistic individuals and neurotypical controls, controlling for the effect of age and sex. Figure S2a: Illustrates the overlap between the group contrast for cortical thickness between autistic individuals and neurotypical controls and the selected 5HT1a receptor density feature map. Figure S2b: Illustrates the overlap between the group contrast for cortical thickness between autistic individuals and neurotypical controls and the selected 5HT4 receptor density feature map. Figure S3: Illustrates the spatial association between the group contrast for cortical thickness between autistic individuals and neurotypical controls and selected feature maps, after controlling for age, sex and age2. Figure S4: Illustrates representative individual maps of deviance from age and sex predicted cortical thickness. Figure S5a: Illustrates the correlation between clinical values (ADI scores) and the spatial association—at the individual level—of cortical thickness deviations with serotonin receptor densities. Figure S5b: Illustrates the correlation between clinical values (ADOS scores) and the spatial association—at the individual level—of cortical thickness deviations with serotonin receptor density. Figure S6: Illustrates the group contrast for cortical thickness between autistic individuals and neurotypical controls, controlling for the effect of age, sex, age2, and after stricter quality control. Figure S7: Illustrates the spatial association between the group contrast for cortical thickness between autistic individuals and neurotypical controls and selected feature maps, after controlling for age, sex, age2, and after stricter quality control. Figure S8: Illustrates the spatial association [file AUR-19-0-s001.docx]

**Supplementary Materials**

**eMethods 1 - Description of Feature Maps**

The D1 receptor surface density map was estimated using D1R-selective radiotracer [^11^C]SCH23390 in PET scans of thirteen healthy volunteers (Kaller et al., 2017). The spatial density of D2 receptors on the cortical surface was assessed by [^18^F]Fallypride binding at PET among 49 healthy participants (Jaworska et al., 2020). The expression of serotonin receptors (5HT1a, 5HT1b, 5HT2a) was estimated by [^11^C]CUMI-101, [^11^C]AZ10419369, and [^11^C]Cimbi-36 binding at PET, among healthy participants (8, 36 and 29 volunteers, respectively) (Beliveau et al., 2017). The spatial density of serotonin receptor 5HT4 was estimated by PET binding of [^11^C]SB207145, in 59 healthy volunteers (Beliveau et al., 2017). 5HT6 was estimated by [^11^C]GSK215083 binding in PET scans, as evaluated in 30 healthy volunteers (Radhakrishnan et al., 2020). Please refer to the original studies for further information about reference maps here included.

**eMethods 2 - Graphical representation of study procedures and analysis workflow.**
a) group-level results; b) individual-level results.

In green, analysis flow for data of the ABIDE dataset.
In blue, external PET data as part of neuromaps toolbox.

Abbreviations: CT = cortical thickness; AI = autistic individuals; TYP = neurotypical controls; Neuromaps = python toolbox to compare brainmaps; D1, D2 = dopamine receptors; 5HT1a, 5HT1b, 5HT2a, 5HT4, 5HT6 = serotonin receptors; mGluR5 = glutamate receptors ADI C = ADI communication; ADI RSI = ADI reciprocal social interaction; ADI RRB = ADI restricted, repetitive and stereotyped patterns of behavior; ADOS C = ADOS communication; ADOS RSI = ADOS reciprocal social interaction; ADOS RRB = ADOS repetitive and stereotyped patterns of behavior.


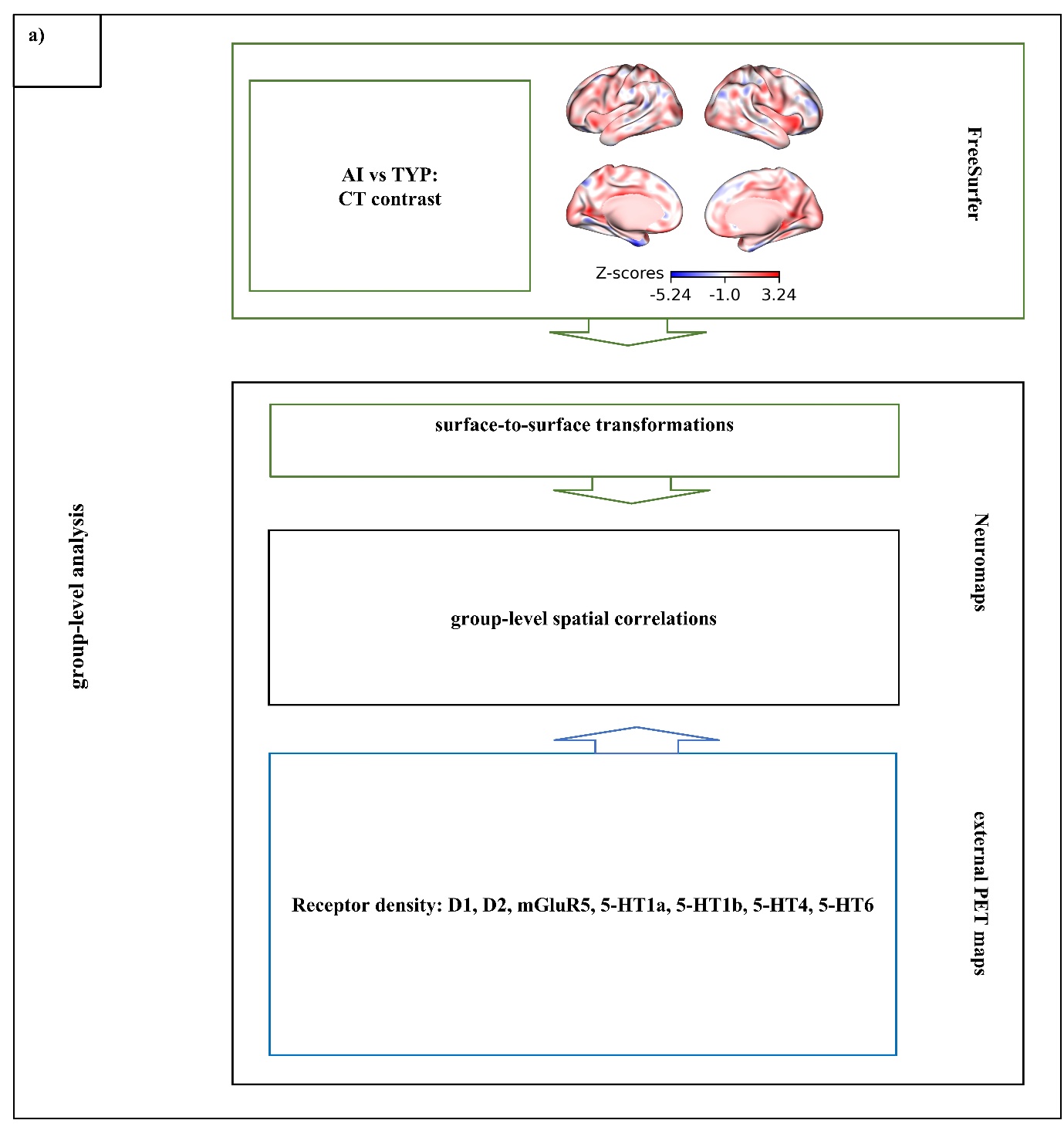


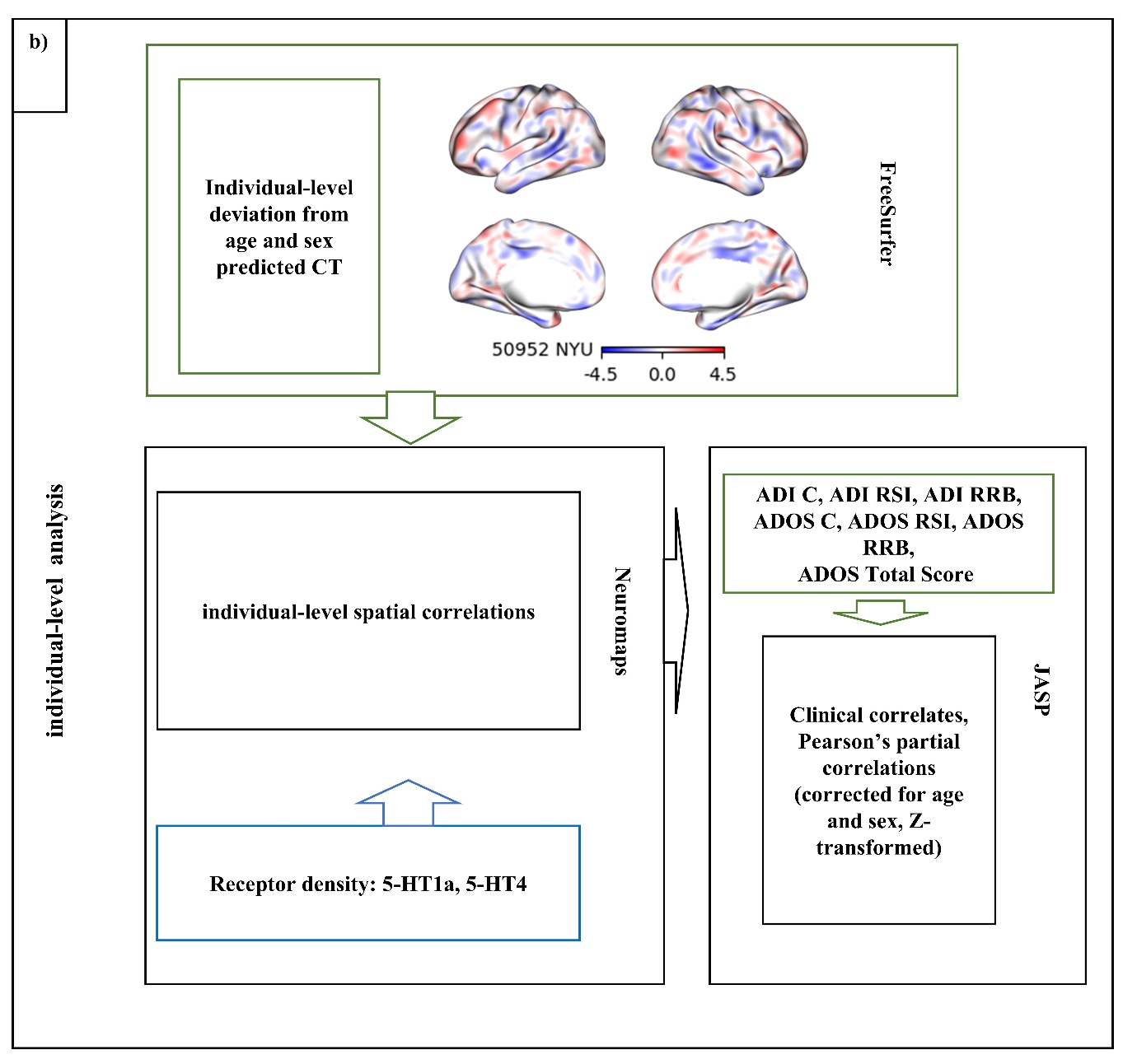


| **Table S1 - Sample Descriptives, sample after stricter MRI quality control (further 115 excluded individuals, 71 autistic individuals, 44 neurotypical controls).** | | | | |
| --- | --- | --- | --- | --- |
|  | **AI** | **TYP** | **U-score /**  **Chi-squared** | **p-value** |
|  | (n = 434) | (n =486) |  |  |
| *Demographics* |  |  |  |  |
| Age  (in years) | 17.58 ± 8.61 | 17.07 ± 7.46 | 106,467 | 0.803 |
| Sex | Males: 375  Females: 59 | Males: 395  Females: 91 | 4.05 | 0.044 |
| BMI | 21.50 ± 5.84 | 19.53 ± 4.54 | 1149 | 0.258 |
| Intracranial volume (mm3) | 1,328,306 ± 263,975 | 1,306,101 ± 240,728 | 111,195 | 0.154 |
| *Psychometric scores* |  |  |  |  |
| ADI C | 15.82 ± 4.68 | / | / | / |
| ADI RSI | 19.76 ± 5.54 | / | / | / |
| ADI RRB | 5.92 ± 2.56 | / | / | / |
| ADOS C | 3.79 ± 1.58 | 0.50 ± 0.61 | 5,584 | <0.001 |
| ADOS RSI | 8.06 ± 2.72 | 0.65 ± 0.81 | 5,728 | <0.001 |
| ADOS RRB | 2.04 ± 1.55 | 0.20 ± 0.70 | 4,210 | <0.001 |
| ADOS Total Score | 11.78 ± 3.78 | 1.15 ± 1.14 | 6,113 | <0.001 |
| *Note*: Mean value ± standard deviation for each variable and study group are shown. As data were not normally distributed, group differences were tested using Mann-Whitney two sample t-tests. As test statistics, U-score, and p-values are stated. Chi-squared was calculated for sex. Autism Diagnostic Interview - Revised (ADI) scores were not available for neurotypical controls. Further details, on subsamples by site of acquisition can be found at [http://preprocessed-connectomes-project.org/abide/](https://fcon_1000.projects.nitrc.org/indi/abide/abide_II.html) .  *Legend*: ADOS = Autism Diagnostic Observation Schedule; AI = autistic individuals; TYP = neurotypical controls. ADI C = ADI abnormalities in communication; ADI RSI = ADI reciprocal social interaction; ADI RRB = ADI restricted, repetitive and stereotyped patterns of behavior; ADOS C = ADOS communication; ADOS RSI = ADOS reciprocal social interaction; ADOS RRB = ADOS repetitive and stereotyped patterns of behavior. | | | | |


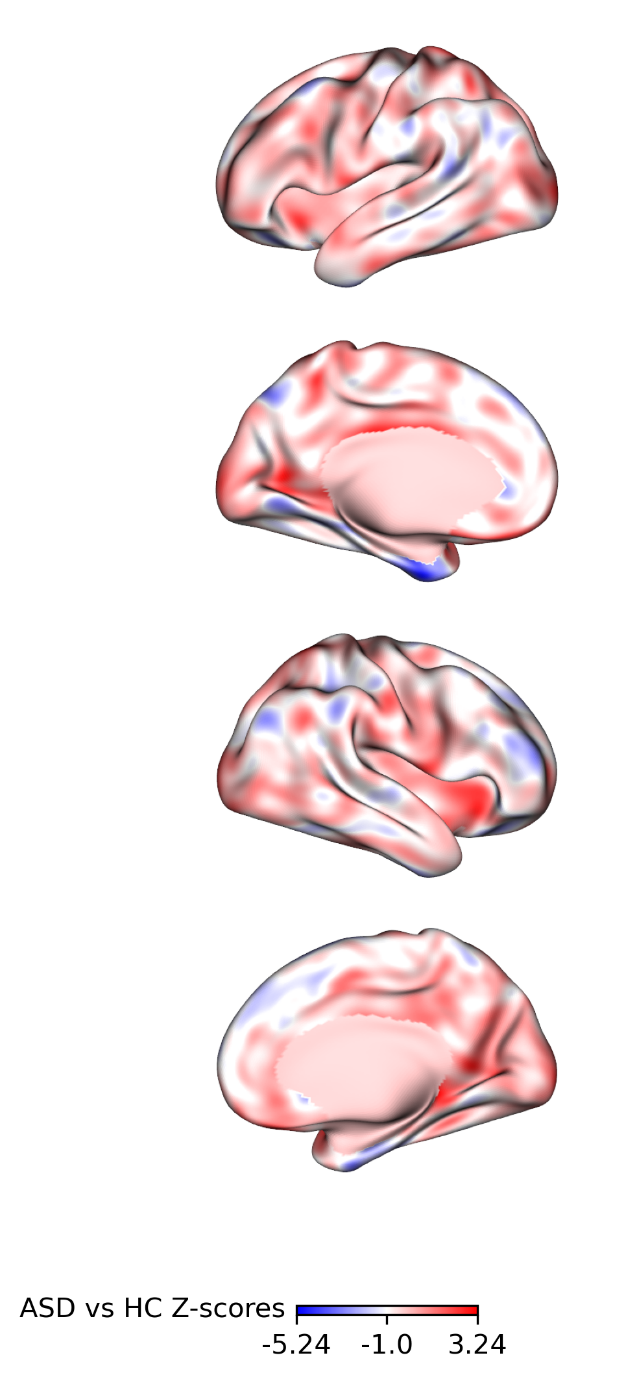


**Figure S1 – Cortical thickness, group contrast between autistic individuals and neurotypical controls, controlling for the effect of age and sex.** Results are not thresholded for statistical significance.
Red = increased CT in autistic individuals. Blue = decreased CT in autistic individuals**.**
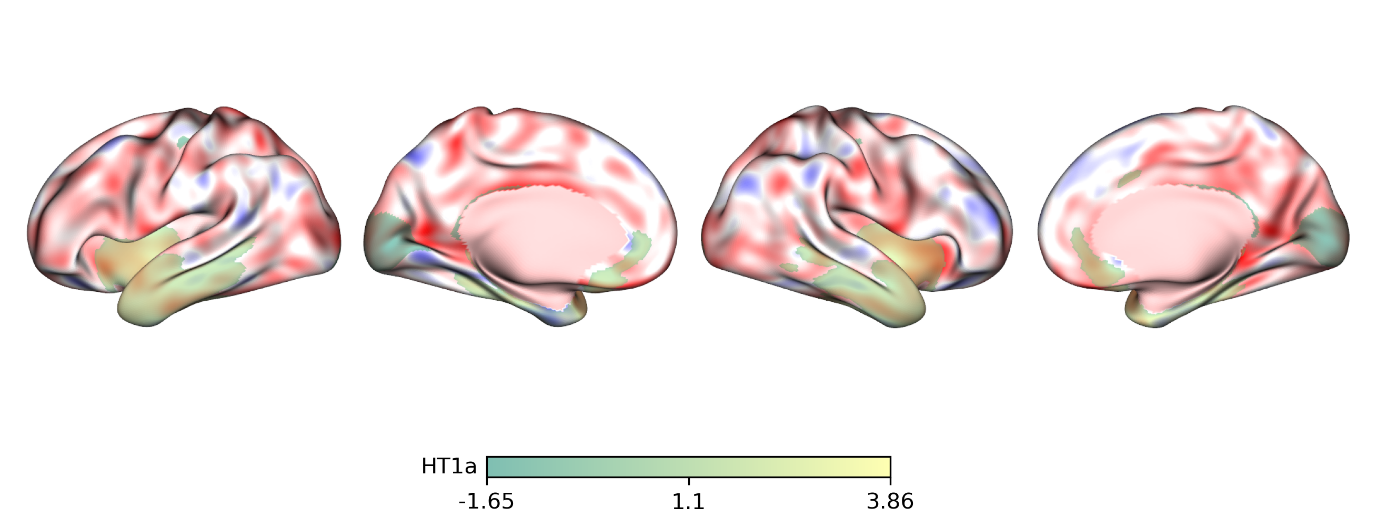

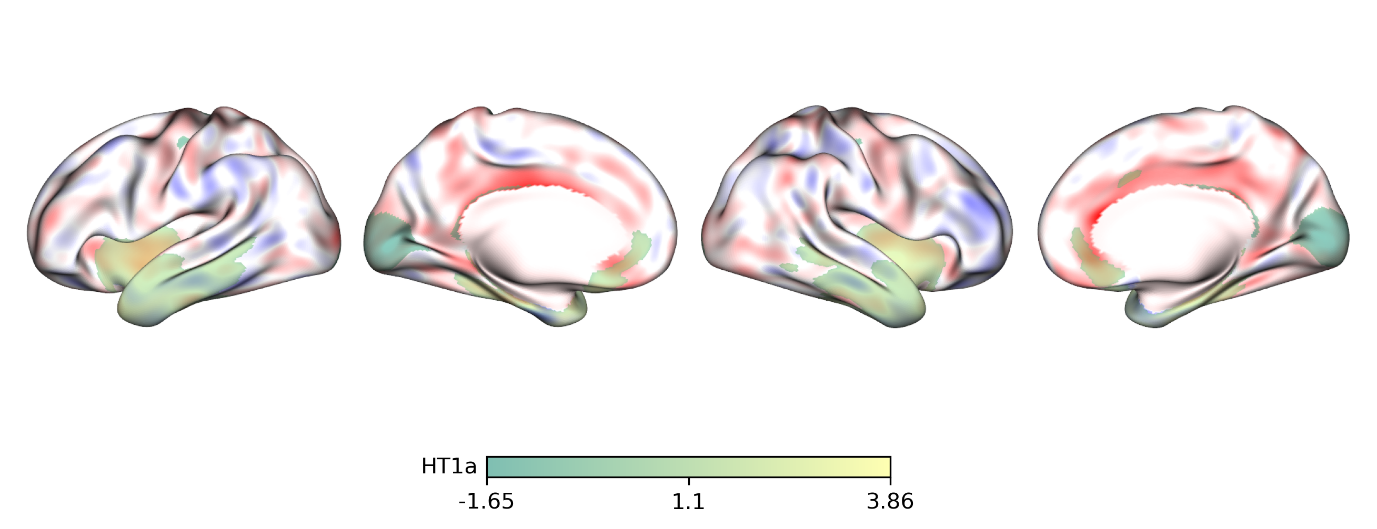
**Figure S2a - Cortical thickness, group contrast between autistic individuals and neurotypical controls; overlap with 5HT1a receptor density.** First, the chemoarchitecture reference feature map was Z transformed and thresholded at Z=1. Next it was binarized and only regions where the thresholded group CT contrast (visualized on a red to blue color scale) and the reference feature map overlapped were depicted. Finally, a minimum cluster size of 5 vertices (as measured on the fslr atlas, 32k).
Above: Results with linear effect of age. Below: Results with linear and quadratic effect of age.

5HT1a

5HT1a

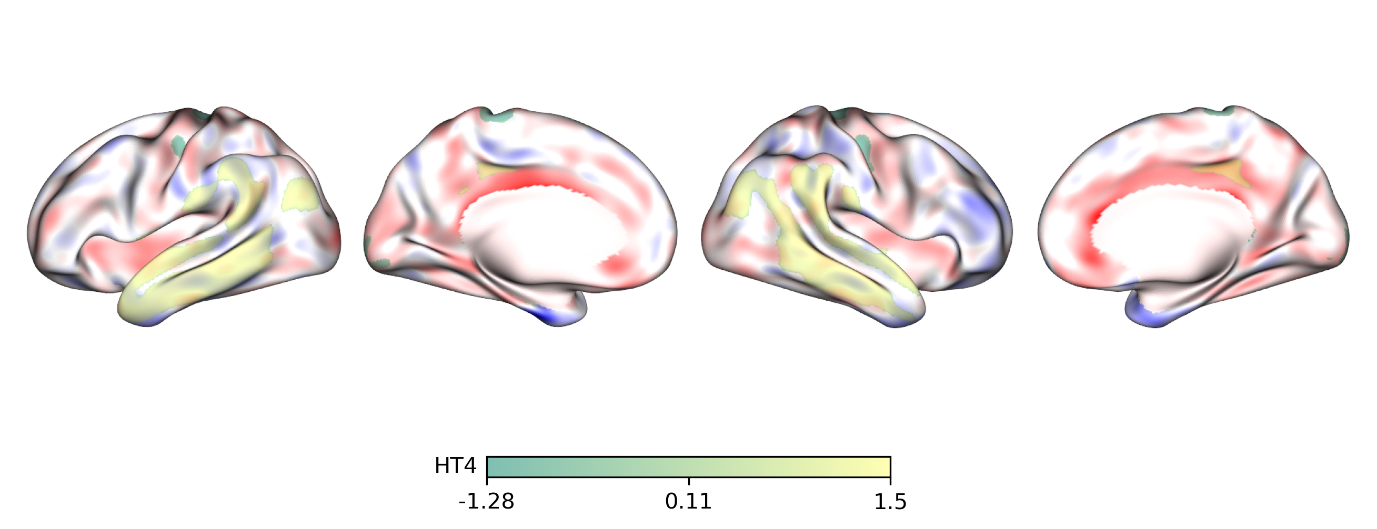

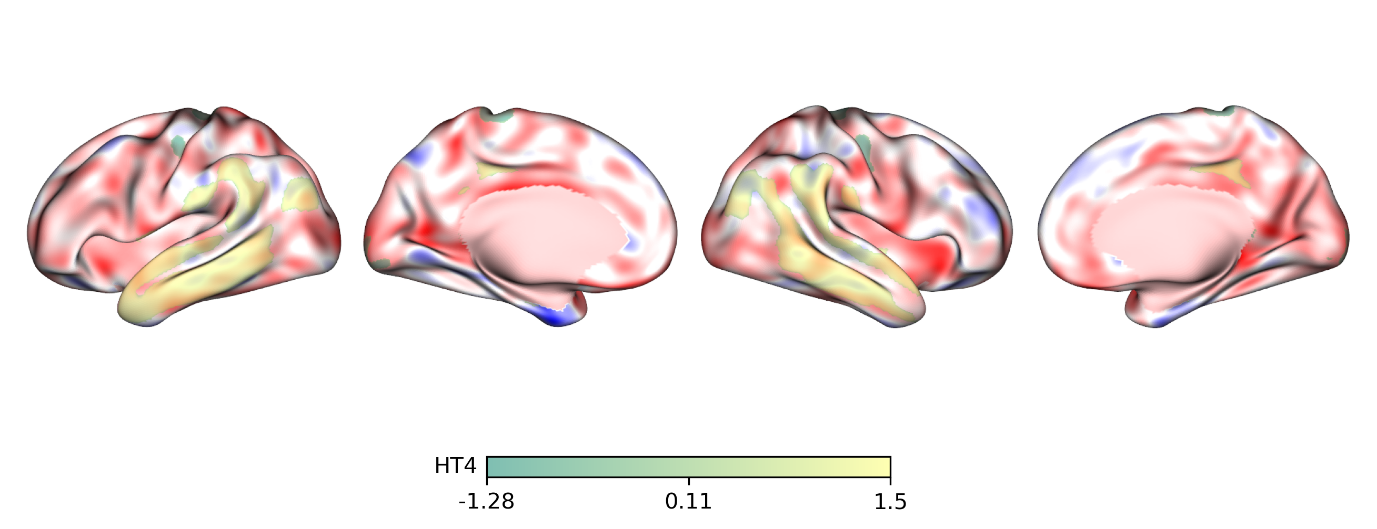


5HT4

5HT4

**Figure S2b - Cortical thickness, group contrast between autistic individuals and neurotypical controls; overlap with 5HT4 receptor density.** First, the chemoarchitecture reference feature map was Z transformed and thresholded at Z=1. Next it was binarized and only regions where the thresholded group CT contrast (visualized on a red to blue color scale) and the reference feature map overlapped were depicted. Finally, a minimum cluster size of 5 vertices (as measured on the fslr atlas, 32k).
Above: Results with linear effect of age. Below: Results with linear and quadratic effect of age.


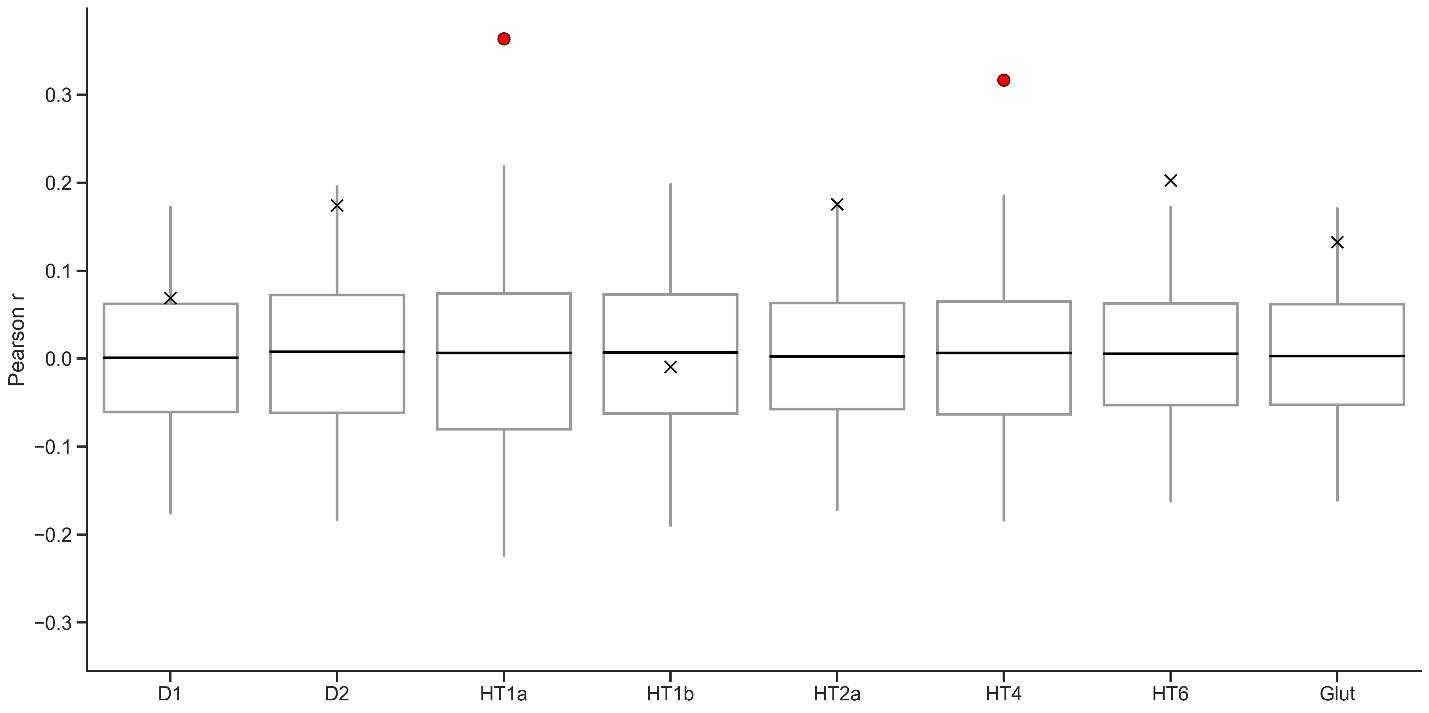
**Figure S3 – Spatial associations of group-level cortical thickness differences with chemoarchitecture features.**Boxplots representing correlation coefficients for rotated images (1.000 permutations), in order to represent 95% confidence intervals of null distributions (BrainSMASH; Burt et al., 2020). The analysis was controlled for the effect of age, sex and age^2^. Empirical results are represented by an “x” if they are not statistically significant, and by a red point if statistically significant (FDR-p < 0.05).

5HT6

5HT2a

5HT4

5HT1b

5HT1a


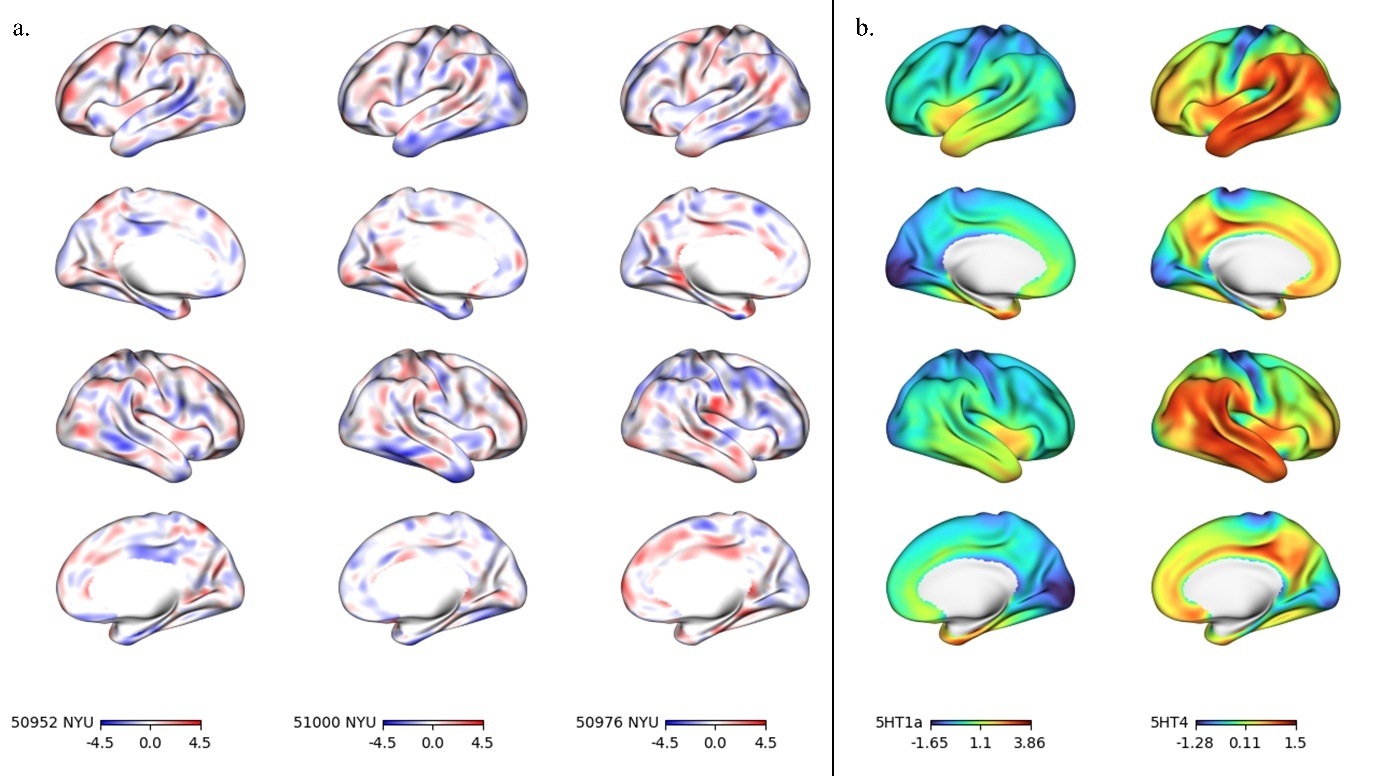


**Figure S4 – Representative individual maps of deviance from age and sex predicted cortical thickness.**On the left, representative individual maps (Z-scored). On the right, selected reference feature maps (Z-scored).
*Note*: reference feature maps and related color maps were adapted from <https://xtra.nru.dk/FS5ht-atlas/>, Center for Integrated Molecular Brain Imaging (CIMBI), Neurobiology Research Unit, Rigshospitalet, Copenhagen University Hospital, Denmark.


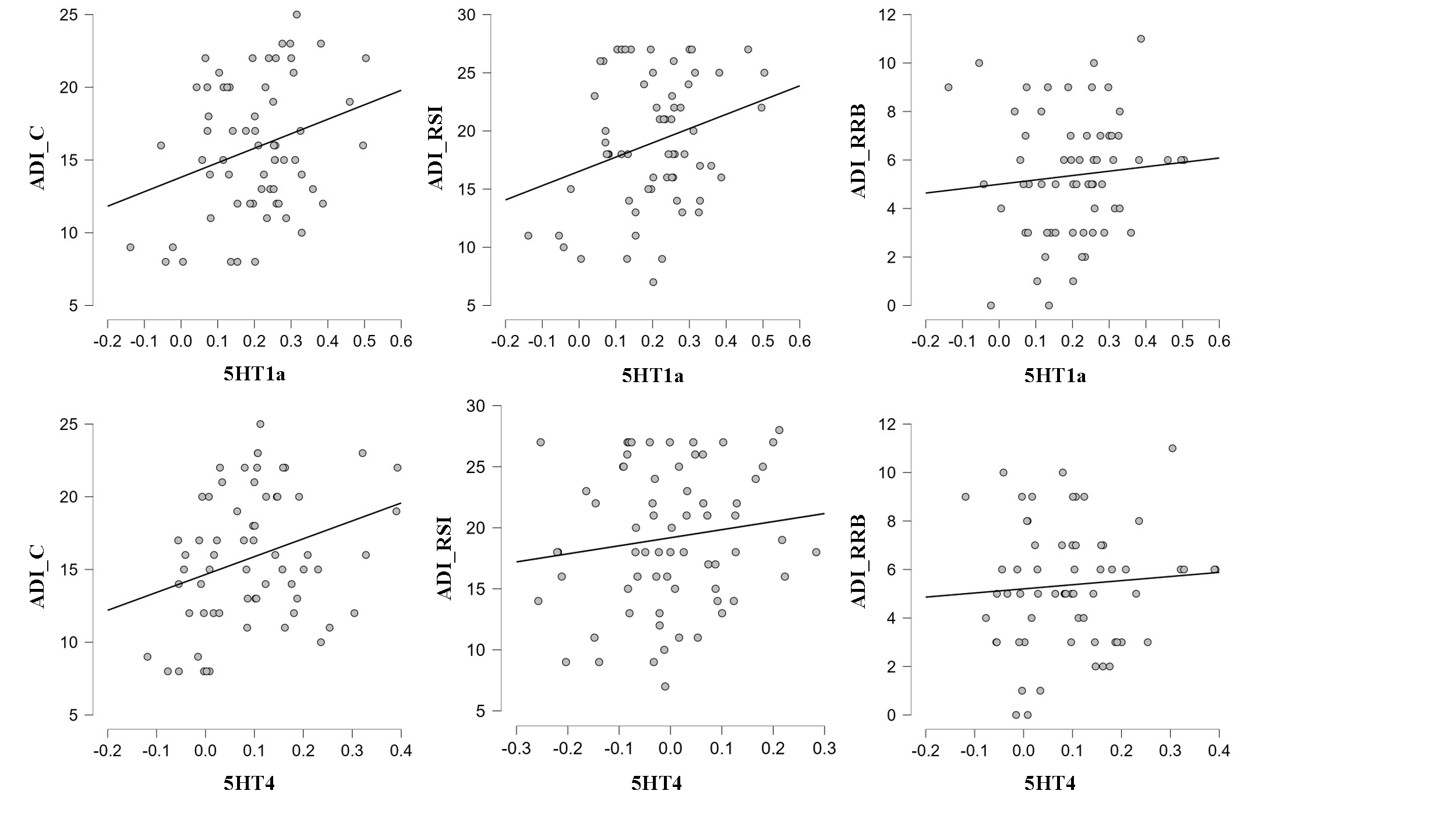


**Figure S5a – Scatterplots depicting correlations between clinical values (Autism Diagnostic Interview - ADI scores) and the spatial association of individual CT deviations with serotonin receptor density.** Greater spatial association between individual CT deviations and serotonin receptor density (x-axis, correlation coefficients after Z-transformation) was linked to higher ADI scores. ADI_C = ADI communication; ADI_RSI = ADI reciprocal social interaction; ADI_RRB = ADI restricted, repetitive and stereotyped patterns of behavior.


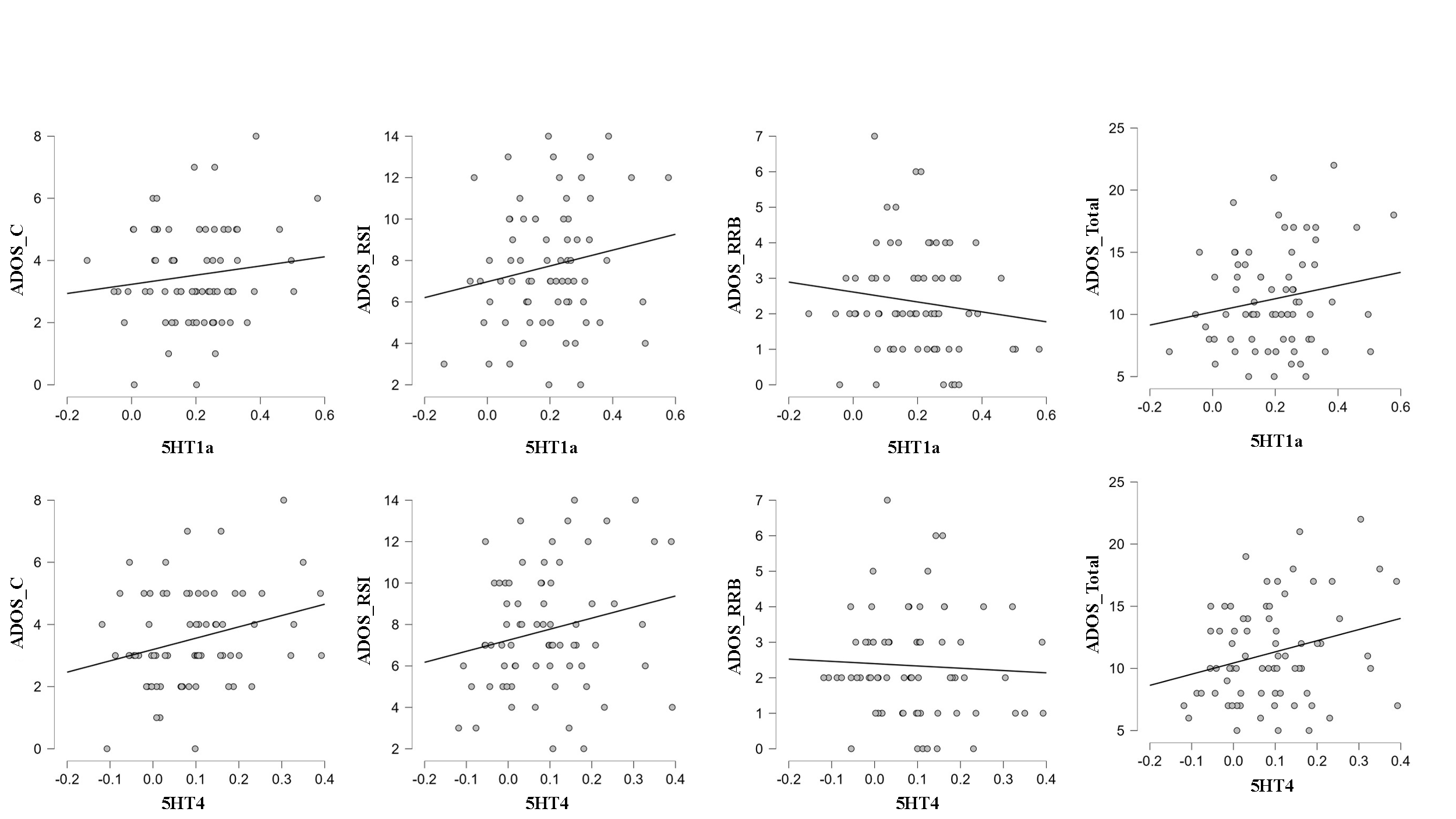


**Figure S5b – Scatterplots depicting correlations between clinical values (Autism Diagnostic Observation Schedule - ADOS scores) and the spatial association of individual CT deviations with serotonin receptor density.** Greater spatial association between individual CT deviations and serotonin receptor density (x-axis, correlation coefficients after Z-transformation) was linked to higher ADOS scores. ADOS_C = ADOS communication; ADOS_RSI = ADOS reciprocal social interaction; ADOS_RRB = ADOS repetitive and stereotyped patterns of behavior


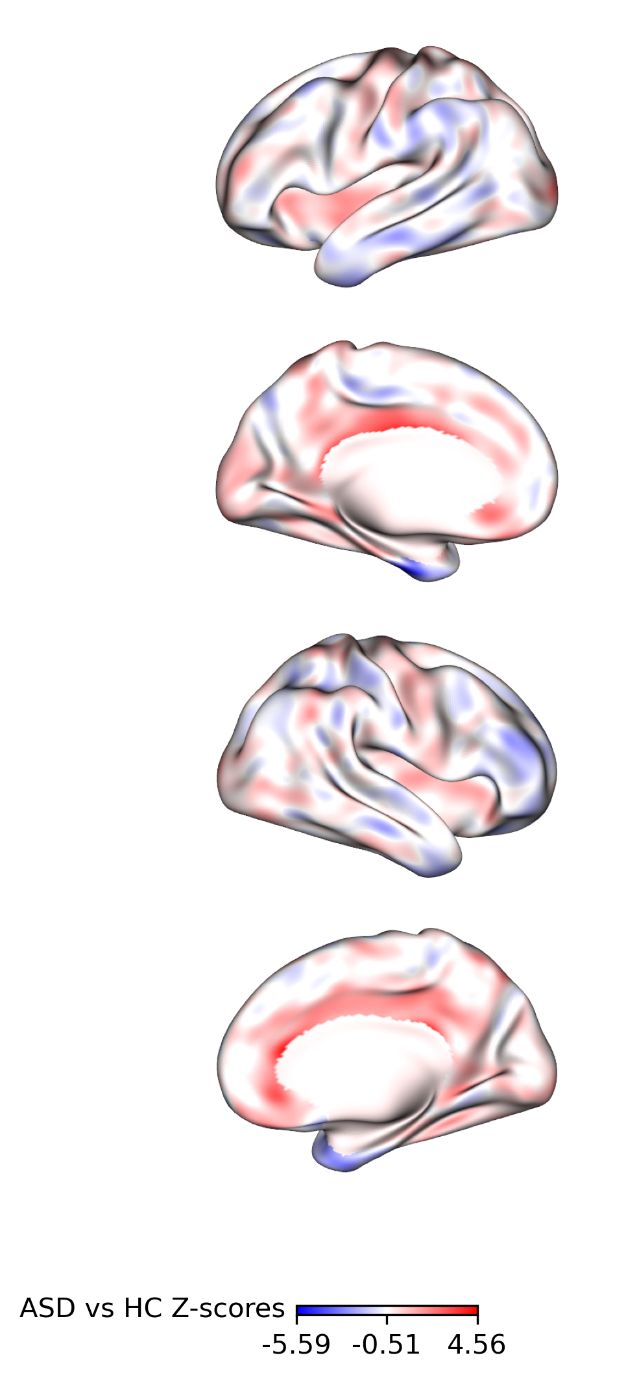


**Figure S6 – Group contrast of cortical thickness (CT) between autistic individuals and neurotypical controls, controlling for the effect of age, age², sex, after stricter quality control which resulted in** **exclusion of 115 participants**. A description of the quality control procedure can be found in the section “Robustness tests” in the methods section of the manuscript. Results are not thresholded for statistical significance. Red = increased CT in autistic individuals relative to neurotypical controls. Blue = decreased CT in autistic individuals relative to neurotypical controls.


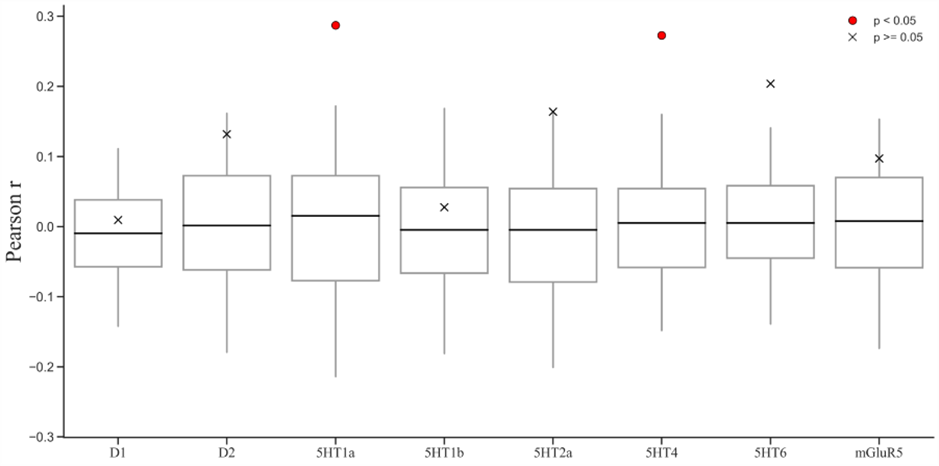


**Figure S7 - Spatial associations of group-level cortical thickness differences with chemoarchitecture features.** This analysis was conducted after the further exclusion of 115 participants due to stricter quality control. The analysis was controlled for the effect of age, sex and age^2^. Boxplots representing correlation coefficients for rotated images (1.000 permutations), in order to represent 95% confidence intervals of null distributions for correlation coefficients (BrainSMASH; Burt et al., 2020). Empirical results are represented by an "x" if they are not statistically significant, and by a red point if statistically significant (FDR-p < 0.05).
*Legend*: dopamine receptors (D1, D2), serotonin receptors (5HT1a, 5HT1b, 5HT2a, 5HT4, 5HT6), glutamate receptors (mGluR5).


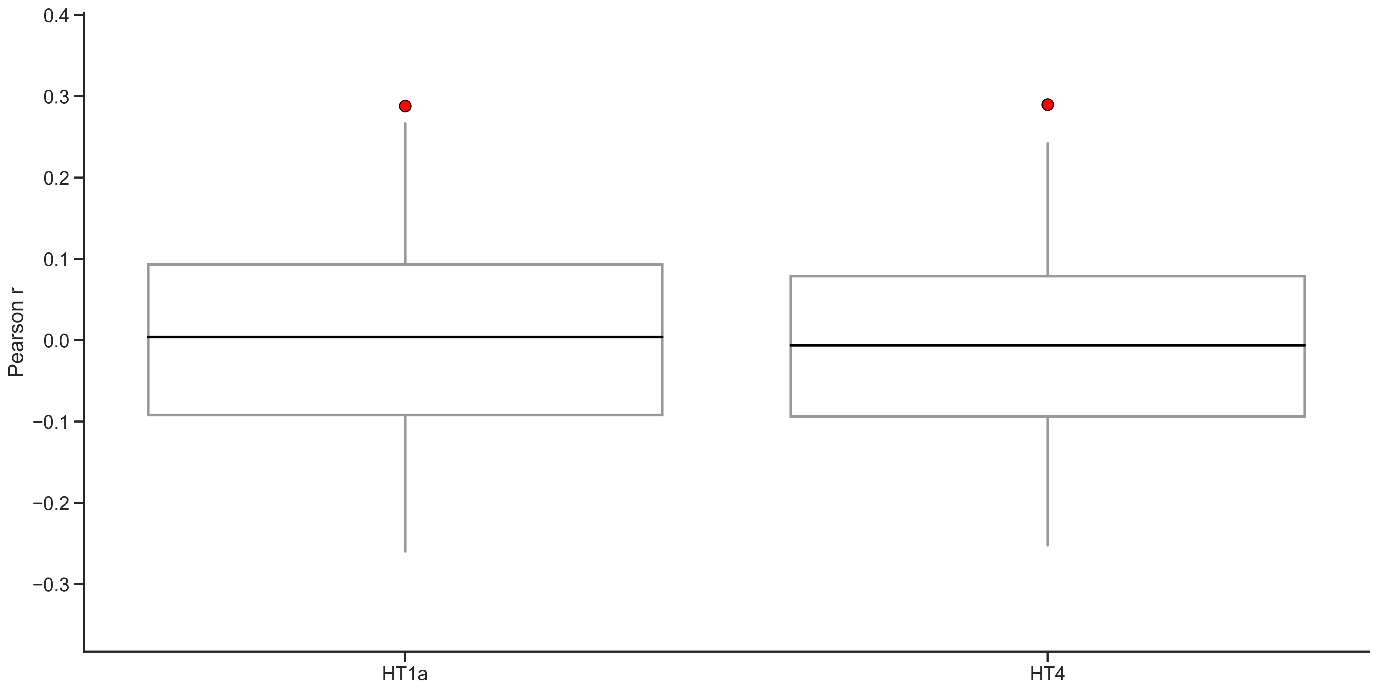


5HT4

5HT1a

**Figure S8 - Spatial associations of group-level cortical thickness differences with chemoarchitecture features.** This analysis was conducted after the further exclusion of 115 participants due to stricter quality control. The analysis was controlled for the effect of age, sex, age^2^ and acquisition site. Boxplots representing correlation coefficients for rotated images (1.000 permutations), in order to represent 95% confidence intervals of null distributions for correlation coefficients (BrainSMASH). Empirical results are represented by a red point if statistically significant (FDR-p < 0.05).
*Legend*: serotonin receptors (5HT1a, 5HT4).

| **Table S2 - Clinical correlates, Pearson’s partial correlations (corrected for age, sex and full IQ)** | | |
| --- | --- | --- |
|  | **5HT1a** | **5HT4** |
| ADI C | 0.254* | 0.274* |
| ADI RSI | 0.256* | 0.273* |
| ADI RRB | 0.086 | 0.069 |
| ADOS C | 0.162 | 0.313** |
| ADOS RSI | 0.161 | 0.206 |
| ADOS RRB | -0.147 | -0.076 |
| ADOS Total Score | 0.178 | 0.206* |
| *Legend*: ADOS = Autism Diagnostic Observation Schedule; ADI = Autism Diagnostic Interview - Revised; ADI S = ADI social interaction; ADI C = ADI communication; ADI RRB = ADI repetitive and stereotyped patterns of behavior; ADOS S = ADOS social; ADOS C = ADOS communication; ADOS RRB = ADOS repetitive and stereotyped patterns of behavior. * p < .05, ** p < .01, *** p < .001 | | |
